# 

**Published:** 1896-07

**Authors:** 


Vol. XVII. JULY, 1896. No. 7.

PUBLISHED MONTHLY AT $3 A YEAR. SINGLE COPIES, 30 CENTS.

### THE JOURNAL OF COMPARATIVE MEDICINE AND VETERINARY ARCHIVES

EDITED AND PUBLISHED BY

RUSH SHIPPEN HUIDEKOPER, Veterinarian (Alfort),

W. HORACE HOSKINS, D.V.S.,

H. D. GILL, V.S.

ALL COMMUNICATIONS AND PAYMENTS SHOULD BE ADDRESSED TO

**Dr. W. Horace Hoskins, Managing Editor, 3452 Ludlow St., Philadelphia, Pa.**

Copies may be had on order of all news-dealers. In London of Baillière, Tindall & Cox.

